# Acknowledgement to Reviewers of *Healthcare* in 2017

**DOI:** 10.3390/healthcare6010004

**Published:** 2018-01-10

**Authors:** Healthcare Editorial Office

**Affiliations:** MDPI AG, St. Alban-Anlage 66, 4052 Basel, Switzerland

Peer review is an essential part in the publication process, ensuring that *Healthcare* maintains high quality standards for its published papers. In 2017, a total of 93 papers were published in the journal. Thanks to the cooperation of our reviewers, the median time to first decision was 26 days and the median time to publication was 54 days. The editors would like to express their sincere gratitude to the following reviewers for their time and dedication in 2017:

Allen, KachinaLophatananon, ArtitayaAllen, Herbert BLu, YingAmiri, AzitaMacMahon, KennethAn, RuopengMacneill, PaulAnsa, BenjaminMagnavita, NicolaAnsa, BenjaminMagnavita, NicolaAnsa, BenjaminMaisha, BuumaAnsa, BenjaminMaltezou, Helen C.Ansdell, GaryMaltezou, Helen C.Antonanzas, FernandoManderscheid, Ronald W.Arnaert, AntoniaMarcus, Esther-LeeAssanelli, DeodatoMarlicz, WojciechAurrekoetxea, Juan J.Masanotti, GiuseppeAydede, Sema KMason, Deanna MarieBailey, CatherineMason, Deanna MarieBasu, PratyushaMasso-Welch, Patricia A.Bathula, ChandraMasterson-Algar, PatriciaBello, AminuMathieu, LucBenetou, VassilikiMazur, JoannaBergbom, IngegerdMcConatha, Jasmin TahmasebBerkowsky, RonaldMcDarby, VincentBerkowsky, RonaldMcKeown, Martin J.Berliner, Janice L.McNaughton, NancyBerry, LoisMearin, FerminBilliot, ShanondoraMiller, JenniferBlack, Helen K.Miller, JessicaBlankestijn, Peter J.Mizuno, KohBodenheimer, ThomasMollica, MichelleBoisen, Kirsten A.Molton, IvanBorovecki, AnaMónica, Perez Bazán LauraBorry, PascalMorano, Kevin A.Bowling, Christopher BarrettMorano, Kevin A.Brand, SergeMorisset, Anne-SophieBreitenfeld Granadeiro, LuizaNaseer, NomanBrett-MacLean, PamelaNeilson, ShaneBrown-Wright, LucyNewman, John F.Bungum, TimothyNewman, John F.Caldeira, SilviaNikolaidis, ChristosCaldeira, SilviaNikolaidis, ChristosCamerino, DonatellaNishi, NobuoCamerino, DonatellaNolan, ClodaghCaserta, DonatellaNorman, RachelCatherine, HayesNourazari, SaraCayón De Las Cuevas, JoaquínNwanodi, OromaChang, Jer-MingO’Connell, RhonaChang, Jer-MingO’Grady, LynChapple, HelenOlson, Michael W.Cheak-Zamora, NancyPagé, GabrielleChristie, DeborahPaludi, MicheleChu, Chun-HungParsi, KayhanChu, Chun-HungPaul-Ward, AmyChua, Tze-ErnPeladic, Nikolina JukicChuang, Kai-JenPeled, RonitClark, H. WestleyPérez-Calvo, Juan I.Clark, Stephanie BrownPeter, RichardClifton, AndrewPiazza, JenniferColver, AllanPiccoli, GBComas, MariaPlateau, CarolynCooper, Sally-AnnPolanska, KingaCoronato, AntonioPolimeni, AntonellaCoyne, ImeldaPollock, Richard L.Cusumano, AnaPorrini, Esteban L.Danielson, Kirstie K.Potvin, NoahDe Gruijl, FrankPoulin, PatriciaDe Perio, MariePoutiainen, AriDe Souza, RussellPruitt, SandiDe Souza, RussellPryss, RüdigerDenham, Bryan E.Quasney, MikeDenner, LarryQuigley, EamonDimitropoulos, GinaRahman, RahbelDominguez, Tyan ParkerRamsey, Vincent K.Dontje, ManonRatnamohan, LuxDos Santos, Eduardo RibeiroRaven, MelissaDryga, AnatolyRay, Robin ADu, K.-L.Reger, MichaelDumler, FReiter, E. MirandaDurbin, JanetRévész, DóraEfird, JimmyRévész, DóraEl Ghoch, MarwanRichmond, JanetErazo, MarciaRigby, Brandon RhettEsposito, CiroRobinson, NedEvans, MaryRodrigues, IsabelFan, JingRoh, Young SookFauchier, Angele CRomeo, GiovannaFerret, RickRuetsch, CharlesFirmino, HoracioRush, Elaine CFord, JonRussell, JoannaFourtounas, CostasSauder, Katherine AnnFourtounas, CostasSawyer, SusanFourtounas, CostasSchalinski, IngaFratkin, MichaelSchmid, ChristopherFreeman, Michael D.Schult, TamaraFriedman, Linda WeiserShafiee, Mohammad AGaultney, JaneShaw, Richard J.Gaynor, KeithShaw, Margie HodgesGerards, SanneShepherd-Banigan, MeganGerdle, BjörnSiega-Riz, Anna MariaGitau, MarySmith, Jared GGitlow, LynnSmith, RobertGoldstein, Rachel RosenbergSnaedal, SunnaGomes, João Fernando PereiraSonestedt, EmilyGoroll, Allan H.Sotiriou, ElenaGoroll, Allan H.Soulage, ChristopheGozal, EvelyneSpagnuolo, RoccoGray, AndrewSpence, MarshaGreaves, LorraineSpilka, JiříGuerrero-Mosquera, CarlosStansfeld, StephenGuevara Lopez, Miguel AngelStanton, TashaGugusheff, JessicaStern, TziporahGuina, JeffreyStins, John F.Gulis, GabrielStricker, RaphaelGulis, GabrielStroumpouki, TheodoraHall, JessicaStylianou, YannisHammill, Bradley GordonSudano, Laura ElizabethHarding, MaireadSukumar, DeepthaHargreaves, DougalTemple, NormanHarpin, ScottThomsen, HaukeHarrington, CharlesThomsen, HaukeHawkins, KeelyTran, Binh Q.Hayward, R. DavidTrevisan, MaurizioHeath, GemmaTrevisan, AndreaHirsch, Annemarie G.Trovik, JoneHo, Roger Chun-ManTso, For YueHowarth, Samuel J.Turner, GillHung, Hsuan-ManVan Ballegooijen, HanneHung, Hsuan-ManVan Gemert, FrederikJacob, JennaVecchio, NerinaJacobsen, RamuneVehkaoja, AnttiJohansen, Guro GravemVenusia, CovelliJyväkorpi, Satu KVenuti, AldoKabir, ThomasVenuti, AldoKagan, Calvin MVolpe, StellaKapellas, KostasVuorio, AlpoKawano, KouichiroWalker, Meghan J.Keida, ElixabethWark, StuartKelley, DarshanWark, StuartKim, Jung YongWatanabe, EiichiKim, Kyung-suWatson, JacquelineKim, Cheol-HoWeiner, Shira SchecterKing, Irena B.Weiner, Shira SchecterKnapp, Caprice A.Wells, YvonneKoehler, KarstenWilkinson, Tom J.Krams, RobWilson, CristinaKrüger, BjörnWiltshire, Esko J.Krysinska, KarolinaWoo, Jessica GrausKurokawa, TetsujiWoodward, AlphaKvaal, KariWu, LiyunLee, Paul H.Wu, LiyunLeggat, Sandra GWu, LiyunLeMasters, TraciWu, LiyunLepp, MargretWu, Ryanne RebekahLesiuk, TeresaWynia, MatthewLewinska, AnnaXu, Feng-LianLewis, Joanne M.Yamamoto, MiwaLewis, Joanne M.Yao, YuChunLiabo, KristinYeh, MayLiapi, CharisYou, ShingchernLiapi, CharisZanutto, AlbertoLilja, MargaretaZhang, BingLin, Yi-ChinZhou, Shang-MingLoi, NatashaZong, GengLophatananon, ArtitayaZuchowski, Jessica L.

